# Dentistry a Specialty of Medicine

**Published:** 1887-03

**Authors:** B. Merrill Hopkinson

**Affiliations:** Baltimore.


ARTICLE II.

### ARE NOT THE ORGANS CONTAINED WITHIN THE ORAL CAVITY PARTS OF THE HUMAN ECONOMY, AND IF SO, HOW CAN THE TREATMENT OF DISEASE IN THIS LOCALITY BE CONSIDERED OTHERWISE THAN AS A SPECIALTY OF THE GREAT HEALING ART, AS COMPREHENDED IN THE TERM: MEDICINE?

BY B. MERRILL HOPKINSON, D. D. S., M. D., OF BALTIMORE.

[Read before the Maryland State Dental Association, February 10, 1887.]

In the January number of the *Independent Practitioner*, published in Buffalo, N. Y., a paper appeared, which had been read before the New England Dental Society, by one of the supposed leading men in our specialty in this country, in which he boldly asserts, "Dentistry is not a Specialty in Medicine." I cannot allow such a statement to go unchallenged, and while I am sure there will be many men far more able than I, who will satisfactorily *answer* his paper, I feel it my duty as a graduate in dentistry and in medicine, as a dentist and as a specialist in medicine, to enter my protest. For *any* individual, be he ever so stupid concerning matters of general education, who had given any time or thought to the relationship between the various branches of medical science, to make such a statement would surprise me, especially if he were to do so after maturing in that portion of his anatomy, he is pleased to call his brain, such ideas as he might have power to bring to bear upon this subject; I say for such an individual to make such an assertion would be a matter of great surprise to me, but, for a man who has the natural ability and talent, to which may be added superior opportunities for advanced study and learning as the gentleman of whom I speak, for such a one to make such a blunder, I can only say that it is most extraordinary and incomprehensible. What can be the reason, in the name of all that is good, for such a course on the part of our distinguished brother? In glancing at his paper I find that he adds only the honored degree of D. D. S., to his name, and the presumption is that he is not a doctor of medicine. Now as a matter of course a man cannot *properly* be a practitioner of medicine or any department thereof, unless he bears the distinctive degree of M. D., and a man is not in the fullest sense a specialist in medicine unless he has legally been made a doctor thereof. This is a positive reason doubtless, why the doctor does not regard himself a specialist in medicine, and so far as he is individually concerned, we will grant him his supposed honor of not being one. Can any man then who is not an M. D. be regarded as a specialist in medicine? Strictly speaking he cannot, but because any individual has not the time or inclination to become an M. D., it does not follow that dentistry in its highest sense and application is not a true department of the great healing art, and I will endeavor to show that it is as much a department of medicine as ophthalmology, otology, laryngology, or any other department, all of which are acknowledged by both profession and laity alike to be specialties. The eminent gentleman should indeed receive the felicit approbation of all men for his happy frame of mind, for while he says he is not in *any sense a specialist* in any science or art, he feels that he is master of a profession far out rivaling all others in magnitude, and scope for doing the greatest good, a connection with which will elevate all who enter its sacred councils, one demanding for its proper practice a knowledge of all the arts and sciences, and more than all, should any other branch of science, more especially medicine, by some happy combination of circumstances become associated with it, such association would be the means of elevating and placing medicine upon the same glorious pedestal where it would shine grandly, but only by reflected light. We congratulate the doctor most heartily and wish him many years of continued and unalloyed happiness in the possession of his *theory* which he so ably advocates, but while we vouchsafe him the above wish, we will endeavor to show that his premises are false and wholly untenable. What is the reason that there should exist at the present time *the occasion* for asking the question, is dentistry a department of medicine? That question can be answered most easily, but in order to do it I must take you back, for a few minutes, almost half a century. There are at this day living witnesses to the fact that before Chapin A. Harris, M. D., founded the Baltimore College of Dental Surgery, he made mighty efforts to have regular chairs of dental surgery established in the faculty of the medical department of the University of Maryland, so that the science and art of dental surgery might not be separated from its parent, medicine, but he made at its birth, so to speak, a special department thereof. Dr. M. A. Hopkinson, who has grown gray in the practice of his chosen profession, was a cotemporary, indeed he was a private student and personal friend of Dr. Harris, and he is my authority for the above statement; indeed the fact is so well known, that I hardly need cite an authority. It is very evident that Dr. Harris recognized the fact, *almost fifty years ago*, that dentistry was a department of medicine, but being denied admission to the medical faculty of the University, and being a man with firm convictions and an established purpose, there was nothing left for him to do but join with his colleagues and found a separate school of learning, the necessity for which he sadly deplored. We will admit that their action and work were noble, that the results have been grand, but who can tell how much grander the results might have been had their first proposition been carried out as intended. What shall be said of the medical faculty of that day? Let us pass their action by in silence, or content ourselves with saying that perhaps the time was not yet ripe; indeed we can truly say the full harvest *is not yet* to be gathered, and *will not be* until the time shall come when there shall be no separate dental schools, but when all men who propose to limit their practice to the diseases of the oral cavity shall be regularly educated as doctors of medicine, which shall be the only degree necessary for all branches, taking at the same time such special instruction as will be needful for them successfully to practice their specialty, and in addition to this, such post graduate courses as may be requisite, doing in fact as other students do, who study and practice other specialties. So much for the original places for which dentistry was intended.

I will venture a prophecy just here, and while I am not gifted in the matter of foretelling coming events as were some of the ancient prophets, I believe I have enough of the gift of prophecy to be able to forecast the coming of such a happy time as I have portrayed concerning the education of our future dental specialist. I sincerely trust that I may live to see the day; if I do, I will with Simeon of old chant my Nunc Dimittis and lie down to rest in peace. I have shown you gentlemen why there exists at this time *the occasion* for a difference of opinion concerning the proper place for dentistry, and we will see if the original idea of its founders, as a science and art, are not as true today as they were nearly fifty years ago. Let us look at the title of my paper. It reads: Are not the organs contained within the oral cavity parts of the human economy, and if so, how can the treatment of disease in this locality be considered otherwise than as a specialty of the great healing art, as comprehended in the term: medicine? How absurd it seems as a matter of fact, *of fact* I say, not because of a semi-established precedent or by force of circumstances, for us to take the organs of incision and mastication, insalivation and deglutition, make a special study of them, combine with this a hurried and perfunctory study of certain other parts of general medicine, add to this a certain amount of mechanical study and manual dexterity, go through the form of a final examination, receive a diploma and then feel that we are prepared to practice a department of the great healing art, in short feel that we can take just as much of the human body as we see fit, study those parts, separate them from the whole, and from a distinct profession! To me there can be nothing more absurd, and such a profession so-called, and so acquired, is to my mind on a par with that of the corn doctor, the pile doctor, and those worthy gentlemen who proclaim to the world their ability to perform all sorts of wonders in the way of curing all diseases of a private and painful nature. Gentlemen, the day for that sort of thing is past and gone, and we should all devoutly thank God for it. The student of this day and generation, in our University Departments at least, is required to have as good an education in anatomy, physiology, materia medica and therapeutics, as the student of general medicine, and he is required as well, to pass a similar examination. What a wonderful stride toward the fulfilment of my prophecy! Far be it from me to wish to derogate one jot or tittle the high standing of my Alma Mater in dentistry, but to show how the curriculum of the student is altered, comparing then with now, I will make a few comparisons. My professor of chemistry in my dental course was also my professor of materia medica and while he was endowed with many characteristics of both scholar and gentleman, he was so unfortunate as to be too fond of the brewings of the jolly god Bacchus; was often absent, and frequently, when present, was not in condition to instruct a class of students. I think I can truthfully say, I did not hear a lecture on materia medica during my dental course, or at least not from that chair, and what I learned of this branch of study was mainly from individual labor. My professor of anatomy was also my professor of physiology, and while he labored faithfully and earnestly to do his duty, a most unprejudiced observer will see that the field is far too vast for one man to accomplish a satisfactory result in either branch. Look with me at these branches as taught in our University. Each one is in the hands of a competent professor, a man standing deservedly high in his special chair and each dental student has the same opportunities as the students in general medicine, and not only has he the same opportunities, but he is obliged at the end of his course of study to be *as proficient.* I speak of these things to show you what a grand advance has been made and to ask you if such a change has taken place in seven years, what may be, nay *what are,* not only the possibilities, but the probabilities, for the succeeding fifty years? I have shown that the fundamental branches of medicine are taught to medical and dental students alike, and I contend that the day *will come* when the *theory* of the principles and practice of dental surgery will be added to the curriculum of the medical student, just as soon as the other branches of general medicine are added to that of the student of dental surgery. I shall be very glad if Dr. Kingsley or any other gentleman will simply answer the question as contained in the title of this paper. To me it appears unanswerable and I cannot understand how the proposition, as expressed, can fail to be subscribed to by all reasonable and sensible men. His paper reads to me like the production of an individual who had been, for some reason or other, snubbed and trodden upon by some weak, bigoted, narrow minded man or men; owning the title of M. D., and I am sorry to say there are many such, and instead of laughing to scorn such silly and puerile attempts at the assumption of greatness, he allows the attack to sink deep into his heart, and as a supposed revenge, vents his spleen upon the whole profession, by seeking to belittle it in the exaltation of his pet, "the dental profession" using shall I call them arguments, to prove that the study and practice of a grand department of the great healing art is a separate and distinct profession; merely by reason of certain fortuitous circumstances, in a more bigoted and less enlightened age, those who had the power and privilege of admitting us to their councils, declined so to do, and forced us to establish schools of our own. I also am proud of my chosen department of practice, proud in the thought of its present exalted position, proud of the good I have accomplished in a modest and quiet way, in the treatment of my fellow beings in distress, and most happy when I look forward and think of the possibility of my accomplishing greater things in the time to come, but I say frankly I was not satisfied with having acquired simply a degree in dental surgery, highly honored as it is, although I labored faithfully and conscientiously during my college course so that in the end I might feel as though I had earned it, and I am sure I derived all the benefit it is possible for a student to derive from such a course of study. I say I was not satisfied, so I immediately began to think of obtaining my degree in medicine, which in due course of time I did obtain from our old and honored University; and in so doing realized all my past ideas of what a true dental specialist should be. There is naturally a division at this time, upon the subject under consideration, and all those interested may be placed in one or the other of the following classes; first, those firmly believing dentistry to be a true department of medicine, viz: graduated doctors in medicine, with perhaps here and there an exception; and second, those equally firmly holding opposite tenets, viz.: non-graduates of medicine; and yet, there are *many* men who occupy positions in the latter category, from force of circumstances, or other reason, who *do* feel that they practice a branch of medicine, for the reason that they fully agree with my proposition, viz.: the title of this paper., In the pursuance of this subject, whatever side we take in the controversy, I beg that, whatever may be said will be devoid of slur and sarcasm, and had Dr. Kingsley omitted much of his article written in this vein, he would doubtless have been read with greater attention and interest. In conclusion, and I fear I have occupied your attention beyond the limit of patient listening, and yet have not said all I desire. I will call your attention to an advertisement upon the last page of the *Maryland Medical Journal*, headed, University of Maryland, School of Medicine. Then follows the word Faculty. Among the list of names, to be found in that faculty, are those of the professors who are specially engaged in teaching those branches pertaining to the study of dental surgery. Why are those names there?

Is it a straw which shows the direction of the whirlwind, the first gentle breezes of which are only now felt, but which in time will come in all its grandeur and might to sweep away all dissension and difference of opinion, driving fıom the minds of those who are unable now to comprehend or grasp the situation, the mist of uncertaınty, which hangs before them like a fog before the eyes, leaving the horizon clear and bright, when will be seen our grand and noble department, not standing alone, but after many years of toil, ever upwards, placed once for all in its unity within the fold, and as a true and worthy child of its rightful parent medicine?—*Maryland Medical Journal.*

